# E3 ligase MKRN2 destabilizes PPP2CA proteins to inactivate canonical Wnt pathway and mitigates tumorigenesis of clear cell renal cell carcinoma

**DOI:** 10.7150/ijbs.107130

**Published:** 2025-08-22

**Authors:** Tiexi Yu, Weiquan Li, Xiangui Meng, Hongwei Yuan, Hailong Ruan, Wen Xiao, Xiaoping Zhang

**Affiliations:** 1 Department of Urology, Union Hospital, Tongji Medical College, Huazhong University of Science and Technology, Wuhan 430022, China.; 2 Shenzhen Huazhong University of Science and Technology Research Institute, Shenzhen 518000, China.; 3 Institute of Urology, Tongji Medical College, Huazhong University of Science and Technology, Wuhan 430022, China.

**Keywords:** Renal cell carcinoma, ubiquitination, E3 ubiquitin ligase, Apoptosis, Wnt signaling pathway

## Abstract

**Background:** Emerging evidence suggests that Makorin Ring Finger Proteins (MKRNs) are dysregulated in various human malignancies. However, the clinical and biological significance of MKRN2 in clear cell renal cell carcinoma (ccRCC) has been minimally explored. In this study, we investigated the exceptional role of MKRN2 in ccRCC.

**Methods:** MKRN2 expression in ccRCC was analyzed with clinical samples and The Cancer Genome Atlas (TCGA) database. The proliferation and migration of cancer cells were assessed by transwell, colony formation, and wound healing assays. Gene expression, DNA methylation, and protein expression and ubiquitination were assessed by real-time PCR, bisulfite sequencing PCR, and western blotting assay, respectively. Protein interactions were verified by co-immunoprecipitation and immunofluorescence assays. *In vivo* experiments identified MKRN2 was a potential tumor inhibitor in ccRCC.

**Results:** Down-regulation of MKRN2 was observed in human ccRCC tissues in both public databases and our clinical samples, mechanistically linked with its promoter DNA hypermethylation. Conversely, overexpression of MKRN2 was associated with ccRCC inhibition and favorable clinical outcomes. MKRN2 interacted with Protein Phosphatase 2 Catalytic Subunit Alpha (PPP2CA) and promoted k48-linked ubiquitination at its K41 residue, leading to the proteasomal degradation of PPP2CA proteins. Consequently, MKRN2-mediated PPP2CA repression increased β-catenin phosphorylation and decreased its protein levels, causing the inactivation of Wnt signaling pathway and amplification of apoptosis in ccRCC cells.

**Conclusions:** This study demonstrated that the E3 ligase activity of MKRN2 had a pivotal role in regulating the PPP2CA-β-catenin-Wnt pathway and granted MKRN2 as a candidate tumor suppressor in ccRCC.

## Introduction

The highly aggressive clear cell renal carcinoma (ccRCC) is the predominant subtype of renal cell carcinomas 1, 2. Currently, surgery is the preferred treatment and the most effective clinical intervention for ccRCC. However, recurrence or metastasis occurs in over 30% of ccRCC patients following surgical intervention 3, 4. Therefore, a deeper understanding of the pathogenic mechanisms underlying ccRCC is urgently needed.

Previous studies have shown that the MKRN family encodes ribonucleoproteins characterized by a unique array of zinc finger domains 5. MKRN2 partially overlaps the RAF1 proto-oncogene in an antisense transcriptional orientation. A defining feature of MKRN2 is a protein-protein interaction motif that is abundant in cysteine and histidine residues, though its specific function remains unknown 6. This motif is common in most E3 ubiquitin ligases, a class of enzymes that transfer ubiquitin from E2 ubiquitin-conjugating enzyme to specific protein substrates. Because ubiquitin ligases either monoubiquitinate or polyubiquitinate of lysine residues through the action of the RING domain 7, 8, certain MKRN2 proteins function as E3 ubiquitin ligases 9. A prior study indicated that MKRN2 acts as a novel E3 ligase that targets the p65 subunit of nuclear factor kappa-B (NF-κB), exerting a negative regulatory effect on inflammatory responses 10. MKRN2 also exhibits anti-tumor action, inhibiting lung cancer development via blocking the PI3k/Akt pathway 11. Additionally, it functions as a tumor suppressor in neurofibromas 12, melanomas 13, and gastrointestinal tumors 14.

The Wnt signaling pathway plays a pivotal role in cellular development, proliferation, differentiation 15-17. Aberrant activation of the Wnt pathway is widely recognized as a significant pathogenic mechanism in several types of tumors 18, 19, including renal carcinoma 20. Under normal conditions, Wnt pathway inhibition is maintained through the phosphorylation and degradation of β-catenin 21. However, β-catenin evades this degradation mechanism in some renal carcinomas, accumulating in cells and then translocating to the nucleus. There, β-catenin prompts aberrant Wnt signaling through genetic mutations or other molecular mechanisms 22.

This study aimed to determine whether MKRN2 was positively correlated with ccRCC prognosis and thus a suitable marker for cancer progression. We demonstrated that elevated MKRN2 expression significantly attenuated ccRCC cell proliferation and metastasis, while inducing ccRCC apoptosis. Mechanistically, MKRN2 triggered K48-linked ubiquitination of PPP2CA at the K41 residue. The resultant degradation and downregulation of PPP2CA augmented N-terminal (S33/S37/T41) phosphorylation and degradation of β-catenin.

## Materials and Methods

### Cell lines and tumor tissues

Human embryonic kidney cells (HEK293T and HEK293), and renal cancer cell lines (A498, 786-O, and Caki-1) were obtained from the American Type Culture Collection (ATCC, Manassas, VA, USA). Cells were cultured in DMEM (Gibco, MA, USA) supplemented with 10% fetal bovine serum (Gibco, MA, USA). Another human renal cell carcinoma cell line OSRC-2 was obtained from National Collection of Authenticated Cell Cultures and cultured in 1640 (Gibco, MA, USA). Human ccRCC tissue samples were collected from Wuhan Union Hospital. All tissue samples were pathologically confirmed to be clear cell renal carcinomas. Research authorization was granted by the Huazhong University of Science and Technology Committee, with explicit written consent obtained from all individuals prior to tissue collection, in accordance with the Declaration of Helsinki.

### Cell infection and transfection

The MKRN2 overexpression plasmid, lentivirus, and their respective controls were procured from GeneChem (Shanghai, China). Small interfering RNA (siRNA) specific to MKRN2 was obtained from GenePharma (Suzhou, China) for MKRN2 silencing. The overexpression plasmids for PPP2CA, PPP2CA-targeted short hairpin RNAs (shRNAs), and the overexpression lentivirus were acquired from GeneChem (Shanghai, China). Lentivirus transfection was performed using the A/P reagent (GeneChem, Shanghai, China), while plasmid, siRNA, and shRNA transfections were carried out with Lipofectamine 3000 (Invitrogen, USA). Transfection procedures adhered strictly to the manufacturer's guidelines.

### Western blotting

Proteins were isolated from ccRCC samples and cells using radioimmunoprecipitation assay (RIPA) buffer supplemented with 1% PMSF and 1% protease inhibitor cocktail. The proteins were separated by SDS-PAGE electrophoresis and transferred onto PVDF membranes (Roche, Basel, Switzerland). To detect target proteins, membranes were incubated overnight with primary antibodies, followed by incubation with secondary antibodies for 1.5 hours. Membranes were washed thorough with PBST to remove unbound antibodies. The full list of the antibodies used was shown in Supplementary Table 1.

### RNA isolation and real-time PCR analysis

Total RNA from ccRCC tissues and cells were extracted with TRIzol reagent (Thermo Fisher Scientific). RNA purity and concentration were assessed using a NanoDrop 2000 spectrophotometer (NanoDrop Technologies, Wilmington, USA). Quantitative real-time polymerase chain reaction (qPCR) using a StepOnePlus™ Real-Time PCR System (Thermo Fisher Scientific, MA, USA) with SYBR Green master mix (Abclonal, Wuhan, China). GAPDH was used as the reference gene for sample normalization, and the gene-specific primers for qPCR are listed in Supplementary Table 2.

### Cycloheximide (CHX) chase assay

Cells were treated with 50 μM CHX (S7418, Selleck, Shanghai, China) for various durations: 0, 4, 8, 12, and 24 hours. After each treatment period, cells were harvested to extract proteins for subsequent western blotting analyses. Protein levels were quantified using ImageJ software. To investigate the protein degradation pathway, specific inhibitors were used: MG132 (HY-13259, MCE, NJ, USA) to inhibit proteasomal activity, and chloroquine (HY-17589A, MCE, NJ, USA) to inhibit lysosomal function.

### Co-immunoprecipitation (Co-IP)

Cell lysates from ccRCC cells were incubated overnight at 4°C with gentle agitation, in the presence of primary antibodies or IgG. The immune complexes were then incubated with protein A/G magnetic beads (#HY-K0202, MCE, NJ, USA) at room temperature for 1.5 hours. After incubation, the beads were washed three times with modified RIPA buffer. The immune complexes separated from the beads were subsequently analyzed by western blot.

### Mass spectrometry (MS) analysis

MS was performed with Genechem (Shanghai, China). Protein gel strips were obtained from co-IP assays involving electrophoresing magnetic immune complexes, then subjected to decolorization, alkylation, enzymatic hydrolysis, extraction, and desalting.

### Subcellular fractionation

Cytoplasmic and nuclear fractions were isolated from ccRCC cells using the Cytoplasmic and Nuclear Protein Extraction Kit (#P0028, Beyotime, Shanghai, China). β-actin was the internal reference for total protein concentrations. Lamin B1 and GAPDH were internal references for nuclear and cytoplasmic proteins, respectively.

### Bisulfite sequencing PCR (BSP)

BSP primer sequences were as follows: MKRN2-BSP-F, 5'- GGATAAGATTGGTAGGATTAAGATGTT -3'; MKRN2-BSP-R, 5'- CACTAACCTACAAATAATCTACTTAATACTCA -3'. Genomic DNA was extracted from ccRCC tissues according to the manufacturer's instructions. After bisulfite treatment, PCR amplification, electrophoresis, and gel purification, the DNA was subjected to transformation experiments using DH5α. Identification was performed by PCR amplification and DNA sequencing.

### Cell viability assay

1,000 cells per well were seeded in a 96-well plate. Cell proliferation was assessed in serum-free medium supplied with 10% of CCK8 reagent (Biosharp, Hefei, China). Cell viability was measured at 0 h, 24 h, 48 h, 72 h, and 96 h.

### Colony formation assay

1,000 cells per well were seeded in 6-well cell culture plate. Colonies were assessed after a 7-day culture period. After the assay, cells were fixed with methanol and viable colonies were identified by staining with 0.05% crystal violet.

### Transwell assay

After 24h of cultivation in a serum-free medium, cells were seeded into the upper chamber of the Transwell. To assess cell invasion, Matrigel was added at a 1:8 ratio. After 24 h, cells were fixed with methanol and stained with 0.05% crystal violet. Images were captured via microscopy, and cell enumeration was performed by randomly selecting multiple areas. The Transwell assay was used to assess both cell migration and invasion capabilities.

### Wound healing assay

Once cells reached 90% confluence in a 6-well plate under serum-free conditions, a wound was created using a 100-µL pipette tip. The wound was washed with PBS to remove debris and images were captured at 0 and 24 h post-injury. Data were analyzed using ImageJ software.

### Flow cytometry apoptosis assay

After treatment, cells were collected and apoptosis was evaluated using a flow cytometer (BD Biosciences, NJ, USA). Prior to analysis, cells were stained with Annexin V-phycoerythrin (PE) and 7-aminoactinomycin D (7-AAD) (#A213-01, Vazyme, Nanjing, China). Results were analyzed using FlowJo software (Becton Dickinson, NJ, USA) to quantify apoptotic events.

### Terminal deoxynucleotidyl transferase dUTP nick end labeling (TUNEL) staining

Cells were first fixed in 4% paraformaldehyde (PFA) for 30 min at room temperature and then treated with 0.3% TritonX-100 for 10 min. After washing with PBS, cells were subjected to staining with a TUNEL detection kit (Beyotime, Shanghai, China).

### Immunofluorescence (IF)

For IF staining, designated cells were cultured on circular slides and fixed with 4% PFA for 20 min. After post-permeabilization with 0.3% Triton X-100, the samples were blocking with 2% bovine serum albumin and incubated overnight with specific antibodies at 4 °C. Next, cells were incubated with the corresponding fluorescently labeled antibodies for 2 h at room temperature, followed by 4,6-diamidino-2-phenylindole (DAPI) staining. Colocalization was assessed under a confocal fluorescence microscope (Nikon Ax, Tokyo, Japan).

### Animal experiments

All animal experiments were conducted with the approval of the Institutional Animal Use and Care Committee at Tongji Medical College, Huazhong University of Science and Technology. BALB/c nude mice were sourced from Beijing HFK Bioscience (Beijing, China). To establish a xenograft tumor model, 2 × 10^6^ A498 cells were transfected with either an overexpression plasmid or a negative control lentivirus, then subcutaneously injected into the axillae of mice. Tumor dimensions were monitored every 5 days for 30 days, when subjects were euthanized. To generate a metastasis model for evaluating metastatic potential, 1 × 10^6^ tumor cells were injected into the tail vein of nude mice. After 60 days, subjects were euthanized to obtain lung and liver metastasis samples for Hematoxylin and Eosin (H&E) staining.

### Immunohistochemical (IHC) assay

For IHC assays, tissue samples were fixed using 4% PFA, dehydration, paraffin embedding, sectioning, deparaffinization, and rehydration, then blocked with bovine serum albumin and incubated with primary antibodies, secondary antibodies.

### Public data and bioinformatics analysis

The Cancer Genome Atlas Kidney Renal Clear Cell Carcinoma (TCGA-KIRC) (version: 2019-07-19) data were downloaded from the UCSC Xena browser for gene set enrichment analysis (GSEA). Bioinformatics tools and networks such as The National Cancer Institute's Clinical Proteomic Tumor Analysis Consortium (CPTAC) and Venny were used for data analysis and visual representations. Statistical and computational analyses were performed in R (version 4.1.3).

### Statistical analysis

Data (mean ± standard deviation, SD) were subjected to normality assessments, Student's t-test or paired Student's t-test, receiver operating characteristic (ROC) curve analysis, Pearson χ^2^-test, and Kaplan-Meier survival analysis with log-rank test. Significance was set at P < 0.05. All statistical analyses were performed in GraphPad Prism 9.0 (GraphPad Software, San Diego, CA, USA) and SPSS 26.0 (IBM Corp., Armonk, NY, USA).

## Results

### MKRN2 is significantly downregulated by DNA methylation in ccRCC and correlated with favorable clinicopathology as well as prognosis

**Figure 1:**
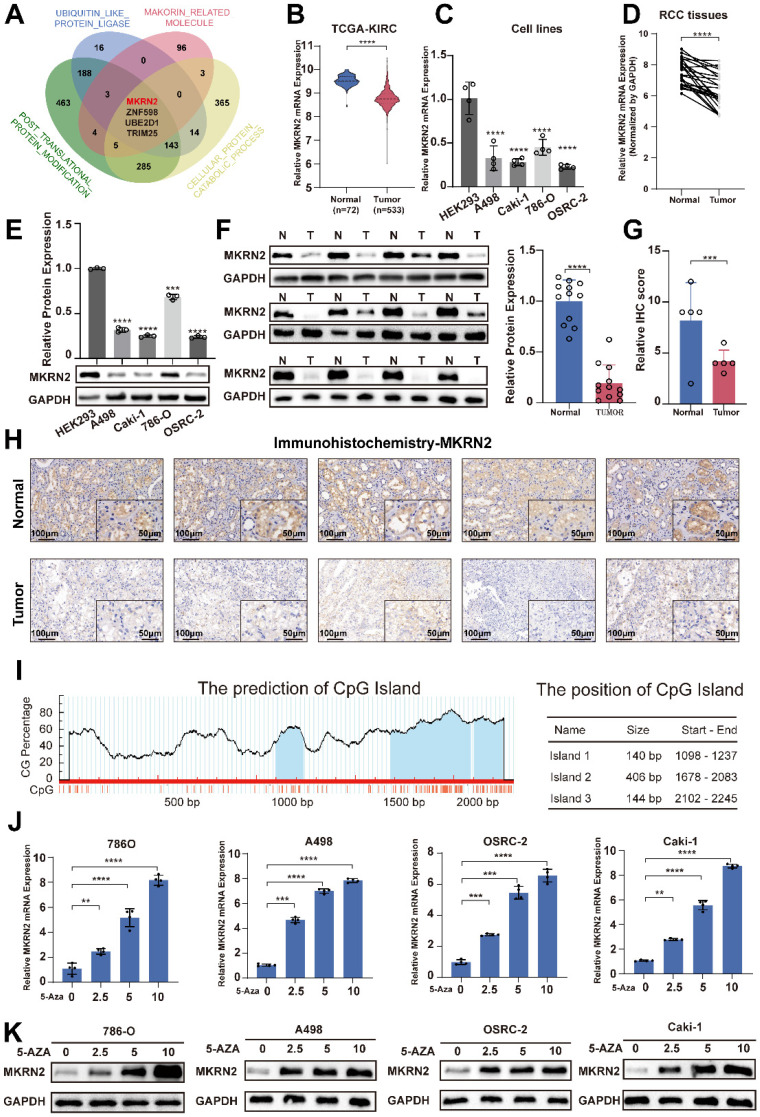
** Epigenetic repression of the MKRN2 gene in ccRCC clinical samples and typical cell lines.** (A) Venn diagram of the Makorin related genes set and three protein modification-related gene sets from the MsSigDB database (https://www.gsea-msigdb.org/gsea/msigdb). (B) The mRNA levels of MKRN2 in ccRCC tissues and paired adjacent normal tissues were based on data from the TCGA (KIRC) database. (C) mRNA levels of MKRN2 in ccRCC cell lines and a normal cell line (n = 4). (D) The mRNA levels of MKRN2 in ccRCC tissues and paired adjacent normal tissues from 24 patients. (E) The protein levels of MKRN2 in ccRCC cell lines and normal kidney cells (n = 3). (F) The protein levels of MKRN2 in ccRCC tissues and paired adjacent normal tissues (n = 12). (G) The relative IHC scores of MKRN2 in ccRCC tissues and paired adjacent normal tissues (n = 5). (H) Representative IHC staining for MKRN2 in ccRCC tissues and adjacent normal tissues from 5 patients. (I) Prediction analysis of CpG islands from the transcriptional start site in the MKRN2 promoter region (http://www.urogene.org/). (J) The mRNA levels of MKRN2 in ccRCC cell lines after 5-AZA treatment (n = 4). (K) MKRN2 protein expression in ccRCC cell lines after 5-AZA treatment. Significance levels denoted as follows: ****p < .0001, ***p < .001, **p < .01, and *p < .05.

We analyzed ubiquitination-related molecules, protein catabolism-associated factors, and post-translational modification genes from the MSigDB database, as well as makorin-related molecules from the GENECARDS database. MKRN2, ZNF598, UBE2D1, and TRIM25 were potential factors influencing renal cancer progression (Fig. 1A). MKRN2 expression was low in ccRCC (Fig. 1B). Kaplan-Meier analysis of TCGA-KIRC data revealed that MKRN2 downregulation was associated with poor overall survival, disease-specific survival, and progression-free interval (Fig. S1G-S1I). In contrast, ZNF598 and TRIM25 were upregulated in tumors, while UBE2D1 expression did not differ (Fig. S1A-S1C) between tumor and adjacent non-tumor tissues; none of these three proteins influenced survival (Fig. S1D-S1F). Additionally, GSE40435 data indicated that MKRN2 was downregulated in renal cancer (Fig. S1J). Furthermore, MKRN2 mRNA (Fig. 1C-1D and Fig. S1K) and protein (Fig. 1E-1F) expression in the four ccRCC cell lines and samples was significantly downregulated from levels in human normal kidney epithelial cells (HEK293) and paired adjacent non-tumor tissues. This result was corroborated with IHC staining, which revealed notably lower MKRN2 protein expression in ccRCC tissues than in adjacent tissues, along with localization predominantly in the cytoplasm (Fig. 1G-1H).

The ROC analysis demonstrated that MKRN2 was a robust discriminator between individuals with ccRCC and healthy controls (Fig. S1L). Bioinformatics analysis of the MKRN2 promoter uncovered a typical CpG island, suggesting that CpG elements may regulate MKRN2 suppression (Fig. 1I) 23. According to BSP, the MKRN2 promoter region experienced significantly elevated methylation in five pairs of renal cell carcinoma tissues and adjacent tissues (Fig. S2A). When we applied methylation inhibitors 5-AZA, MKRN2 mRNA and protein expression both increased in ccRCC cell lines (Fig. 1J-1K). Taken together, these results indicates that DNA methylation profile of MKRN2 promoter may pre-deposit its gene expression and ccRCC prognosis.

### MKRN2 promoted cell apoptosis, suppressed proliferation and metastatic potential of ccRCC

**Figure 2:**
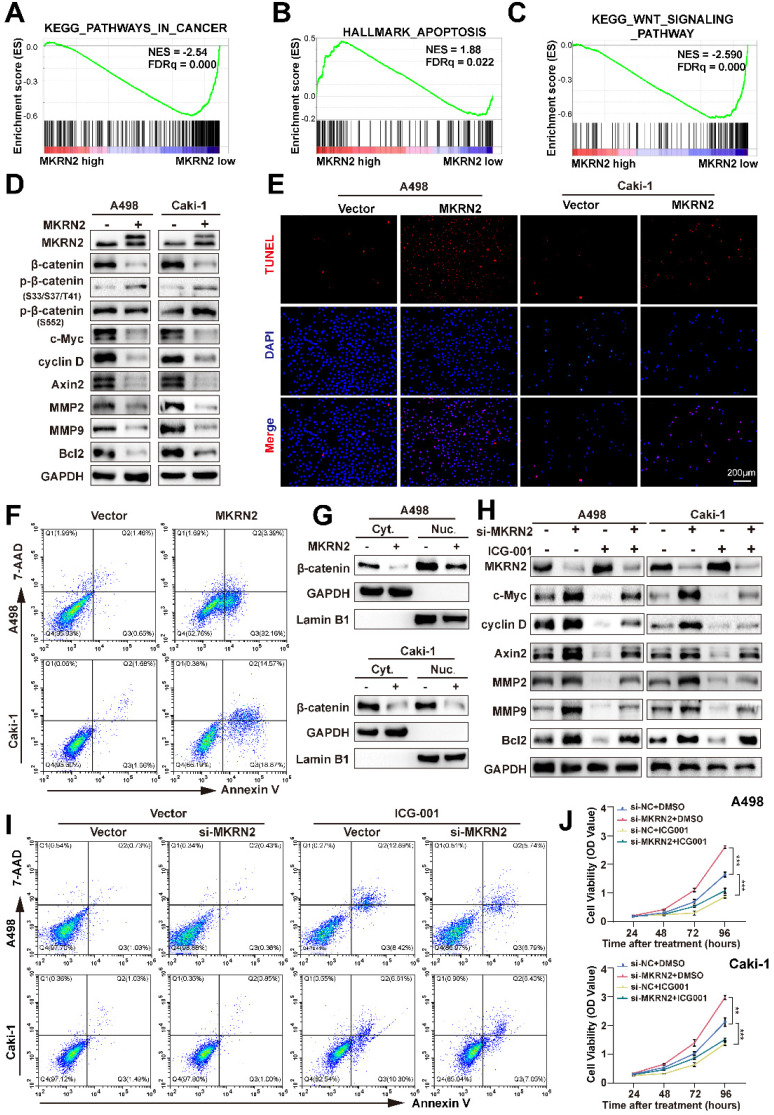
** MKRN2 inactivates the canonical Wnt pathway and motivates ccRCC cell apoptosis.** (A, B, C) Gene Set Enrichment Analysis (GSEA) revealed significant associations between pathways in cancer, apoptosis, and Wnt signaling pathways in ccRCC and the mRNA levels of MKRN2. A False Discovery Rate (FDR) of less than 25% was considered statistically significant. (D) Western blot assay was conducted to evaluate the protein levels of Wnt and apoptosis-related proteins in MKRN2 overexpressing cells and the control. (E) TUNEL staining was employed to illustrate the rate of apoptosis in the indicated cells. (F) Flow cytometry analysis was employed to determine the proportion of apoptotic cells in samples with MKRN2-OE compared to control cells. (G) Western blotting shows subcellular localization of β-Catenin after MKRN2-KD. (H) Western blotting shows protein levels of MKRN2 and Wnt related-genes in the indicated cell lines. Cells with MKRN2-KD were treated with 10 µM ICG-001 (an inhibitor of β-catenin/TCF) or DMSO. (I) Flow cytometry analysis was employed to determine the proportion of apoptotic cells in indicated cell lines. (J) CCK8 assays for indicated cell lines (n = 4). Significance levels denoted as follows: ****p < .0001, ***p < .001, **p < .01, and *p < .05.

GSEA test revealed the associations of MKRN2 with tumorigenesis (Fig. 2A), apoptosis (Fig. 2B), and the Wnt signaling (Fig. 2C) in ccRCC. Next, we found that in renal cancer cells, overexpression of MKRN2 (Fig. S3A and S3B) led to elevated β-Catenin phosphorylation and reduced β-Catenin protein expression, resulting in the attenuation of Wnt pathway that is marked by the down-regulation of signature genes (c-Myc, Cyclin D, Axin2, Bcl-2, MMP2, and MMP9) (Fig. 2D and Fig. S5A, S5B). In comparison, MKRN2 knockdown (KD) enhanced the expression of these aforementioned molecules, together with the decrease in β-Catenin phosphorylation (Fig. S5C-S5E).

TUNEL fluorescence staining and flow cytometry suggested that MKRN2 overexpression (OE) increased apoptosis in renal cancer cells (Fig. 2E-2F and Fig. S4C-S4D), while significantly suppressing proliferative capacity (Fig. S3E-S3G), cell invasion and migration (Fig. S3I), and wound-healing capacity (Fig. S4A) in A498 and Caki-1 lines. MKRN2-KD had the opposite effect, promoting proliferative capacity (Fig. S3F and S3H), cell invasion and migration (Fig. S3J), and wound-healing capacity (Fig. S4B).

Nucleocytoplasmic separation assays revealed that MKRN2-OE occurred concurrently with β-catenin downregulation in both the cytoplasm and nuclei of renal cancer cell lines (Fig. 2G). The β-catenin inhibitor ICG-001 did not affect MKRN2 protein expression, further validating the pivotal role of Wnt signaling in MKRN2-mediated apoptosis and tumor suppression (Fig. 2H). Western blotting revealed significant c-Myc, cyclin D, axin2, and Bcl-2 downregulation in MKRN2-KD cells upon β-catenin inhibition (Fig. 2H). Additionally, β-catenin inhibition significantly increased apoptosis (Fig. 2I and Fig. S4G), and decreased proliferation (Fig. 2J), invasion, and migration (Fig. S4E-S4F) of MKRN2-KD cells. These findings are in line with the notion that MKRN2 deactivates the Wnt pathway to promote an apoptosis-prone phenotype, impeding ccRCC progression.

### PPP2CA promoted the progression of renal cancer via the Wnt signaling pathway

**Figure 3:**
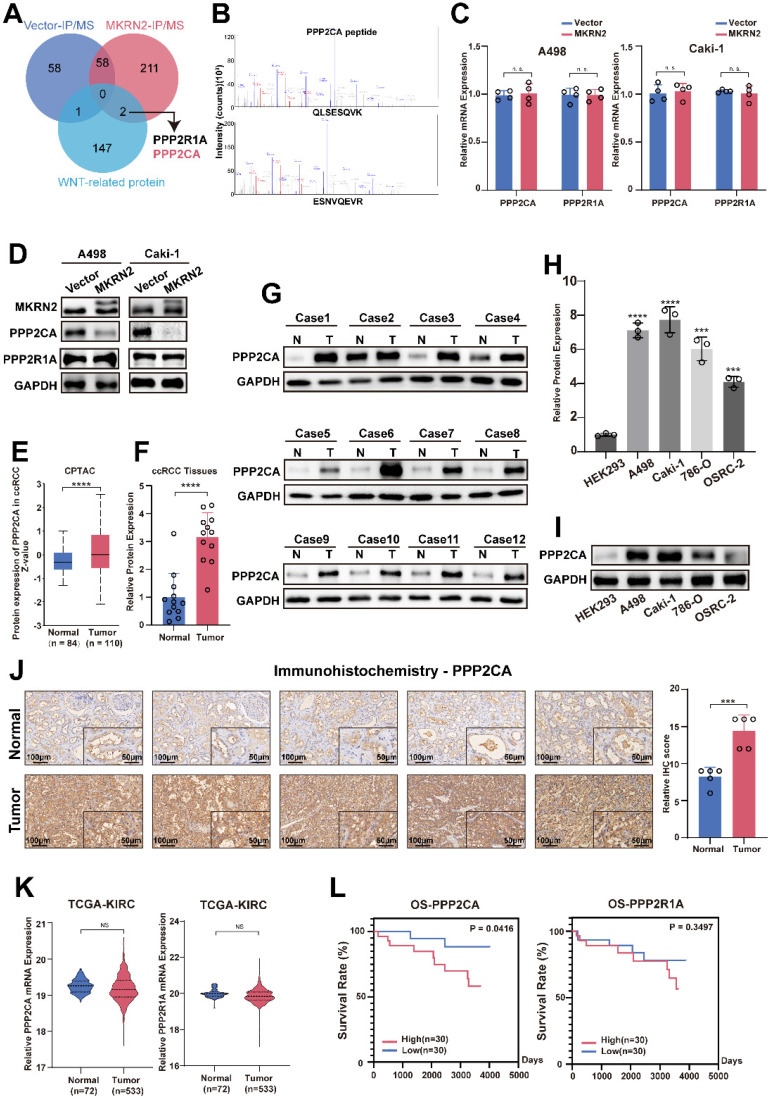
** Identification of PPP2CA in the MKRN2 protein complex that is over-expressed in ccRCC.** (A) A Venn diagram illustrates proteins that interacted with Wnt-related molecules (KEGG_WNT_SIGNALING_PATHWAY), proteins pulled down via Flag-MKRN2, and proteins pulled down via Vector in A498 cell lines. (B) PPP2CA peptides that were pulled down by Flag-MKRN2. (C) the mRNA expression level of PPP2CA and PPP2R1A in MKRN2-OE renal tumor cell lines (n = 4). (D) Western blot analysis was employed to confirm the two potential targets of MKRN2. GAPDH served as a loading control. (E)PPP2CA protein expression was elevated in renal cancer tissues compared to adjacent non-cancerous renal tissues. (F) and (G) The protein levels of PPP2CA were evaluated in 24 ccRCC tissues and adjacent nonmalignant tissues. (H) and (I) The mRNA and protein levels of PPP2CA were assessed in RCC cell lines (786-0, A498, Caki-1, and OSRC-2) and a normal cell line (293). (J) Immunohistochemistry (IHC) staining was performed for PPP2CA in ccRCC tissues and adjacent nonmalignant tissues. (K) No significant differences in the mRNA expression levels of PPP2CA and PPP2R1A in the TCGA-KIRC database. (L) Survival analysis showed a significant difference for PPP2CA with no significant difference in PPP2R1A from the CPTCA database. (Significance levels denoted as follows: ****p < .0001, ***p < .001, **p < .01, and *p < .05.

We conducted differential protein analysis using Co-IP and protein profiling to further explore the effect of MKRN2 on the Wnt pathway in ccRCC. The results indicated that both PPP2CA and PPP2R1A proteins were associated with the Wnt signaling pathway (Fig. 3A-3B), but their RNA expression was not (Fig. 3C). When MKRN2 was overexpressed, PPP2CA protein levels decreased, but PPP2R1A protein levels did not (Fig. 3D). Data from CPTAC showed that PPP2CA protein expression was higher in renal cancer tissues than in adjacent non-cancerous renal tissues (Fig. 3E), corroborating our experimental data showing significantly higher PPP2CA protein concentrations in ccRCC tissues (Fig. 3F-3G) and other renal cancer cell lines (Fig. 3H-3I) than in normal tissues. Likewise, IHC analysis revealed significantly elevated PPP2CA protein expression in ccRCC tissues compared with adjacent regions (Fig. 3J).

We did not observe significant differences in PPP2CA and PPP2R1A mRNA expression from TCGA-KIRC (Fig. 3K). Survival analysis performed on CPTCA data found a significant effect for PPP2CA but not for PPP2R1A (Fig. 3L). Therefore, we established PPP2CA-OE and PPP2CA-KD renal tumor cell lines (Fig. S6B). Notably, CCK8 and colony formation assays revealed that PPP2CA downregulation led to a significant reduction in the proliferative capability of ccRCC cells (Fig. S6C-S6D and S6F). In contrast, PPP2CA-OE enhanced renal cancer cell migration, invasion, wound healing, and colony formation (Fig. S6E, S6G-S6I).

### MKRN2 inhibited ccRCC progression and promoted apoptosis through PPP2CA mediated-suppression of Wnt signaling pathway *in vitro*

**Figure 4:**
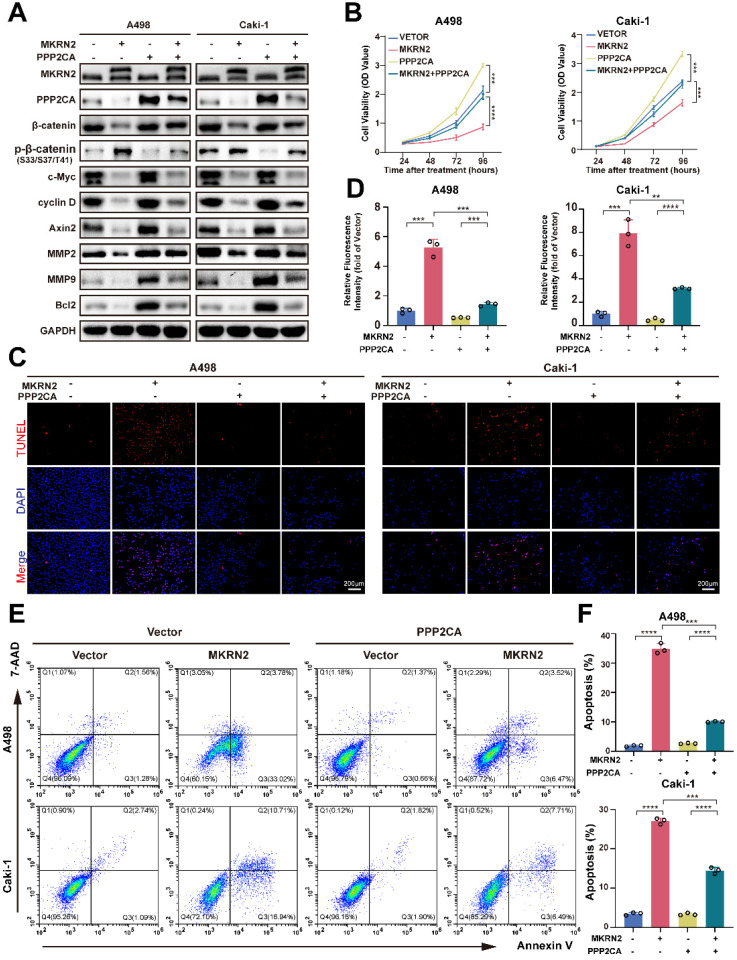
** MKRN2 represses ccRCC proliferation and promoted tumor cell apoptosis by antagozing PPP2CA-activated Wnt pathway.** In light of the overexpression of MKRN2, cellular models featuring concurrent overexpression of PPP2CA were generated via the transfection of PPP2CA lentivirus. (A) A Western blot assay was employed to evaluate the protein levels of MKRN2 and PPP2CA in the specified cells. (B) Cell growth curves were established through CCK8 assays for the designated cells (n = 3). (C, D) TUNEL staining was employed to illustrate the apoptosis rate in the designated cells (n = 3). (E, F) Flow cytometry analysis was employed to ascertain the proportion of apoptotic cells in the specified samples (n = 3). Significance levels denoted as follows: ****p < .0001, ***p < .001, **p < .01, and *p < .05.

MKRN2 and PPP2CA protein concentrations were negatively correlated in ccRCC (Fig. S6A). To better understand this MKNR2-PPP2CA axis, we established PPP2CA and/or MKRN2-OE cell lines. Our analysis demonstrated that PPP2CA-OE abolished MKRN2 inhibition in the Wnt signaling pathway (Fig. 4A). Furthermore, CCK8 and colony assays revealed that PPP2CA upregulation partially alleviated the inhibitory impact of MKRN2 on cell proliferation (Fig. 4B and Fig. S7C-S7E), migration, and invasion (Fig. S7A-S7B and Fig. S7F-S7H), substantially reversing MKRN2's biological effects (Fig. 4C-4F). MKRN2-OE alone led to a significant increase in apoptosis, whereas PPP2CA-OE alone partially diminished apoptosis. In summary, MKRN2 suppresses ccRCC progression, primarily influencing tumor cell apoptosis via PPP2CA-mediated Wnt signaling.

### MKRN2 targeted PPP2CA for its proteasomal degradation

**Figure 5:**
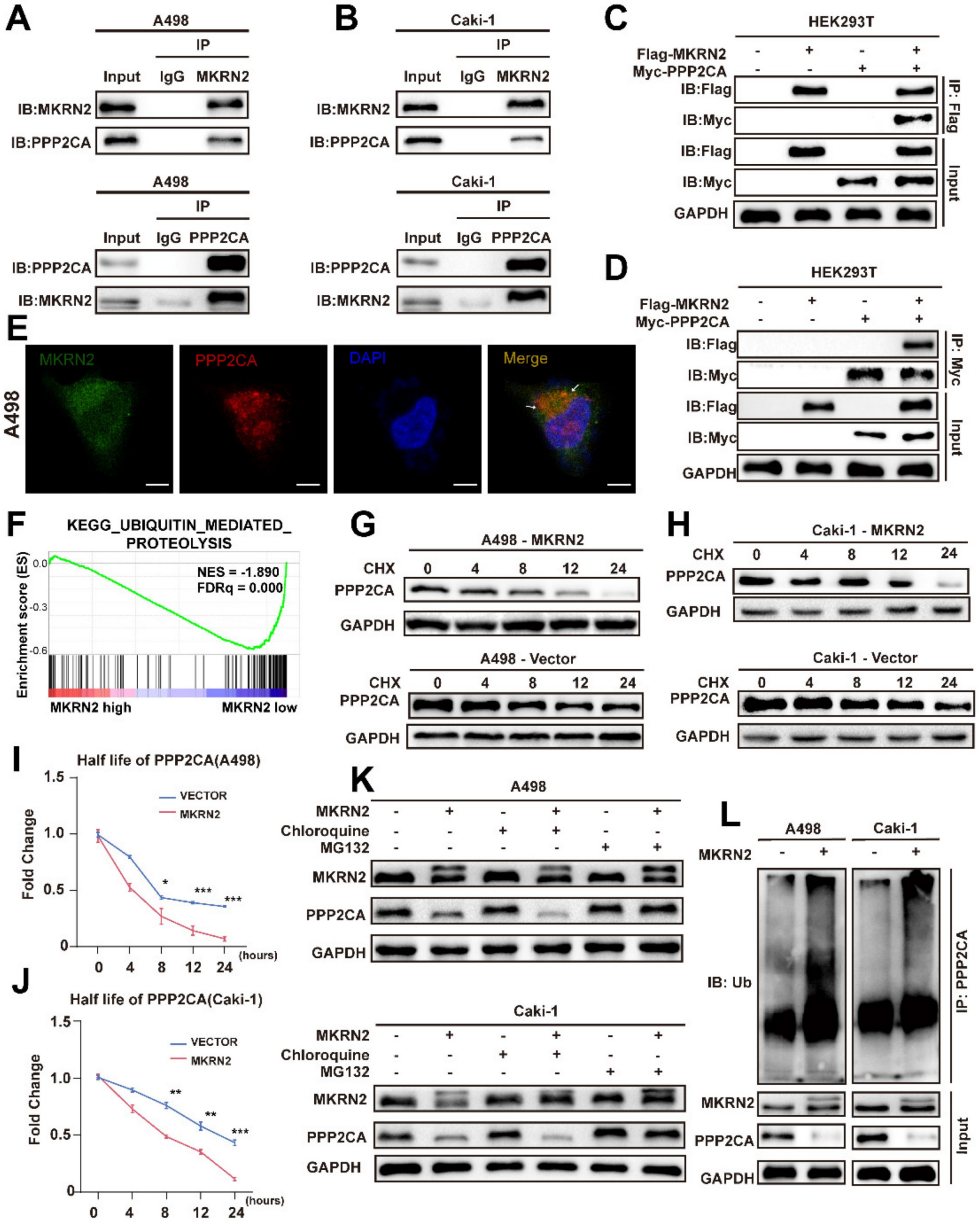
**MKRN2 interacts with and destabilizes PPP2CA protein by catalyzing its poly-ubiquitination that is coupled to proteasome-specific degradation pathway**. (A, B) Co-immunoprecipitation (Co-IP) assays were conducted to evaluate the endogenous interaction between MKRN2 and PPP2CA in A498 and Caki-1 cells. (C, D) Co-IP assays were also employed to determine the exogenous interaction between MKRN2 and PPP2CA in HEK293T cells overexpressing Flag-MKRN2 and/or Myc-PPP2CA. (E) Representative confocal microscopic images illustrate the colocalization of MKRN2 and PPP2CA in A498 cells. (F) GSEA revealed significant associations between ubiquitin-mediated proteolysis and the mRNA levels of MKRN2. (G, H) ccRCC cells with stable MKRN2-KD and OE were subjected to cycloheximide (CHX) treatment at specified time intervals. Subsequently, cells were collected, and PPP2CA protein expression was assessed via western blotting (n = 3). (I, J) Quantitative analysis was performed to determine the relative protein level of PPP2CA in (G, H). (K) ccRCC cells with stable MKRN2 -OE were treated with vehicle (DMSO), MG132 (20 × 10-6 M), or chloroquine (50 × 10-6 M) for 12 hours. Western blot analysis was employed to evaluate the protein level of PPP2CA. (L) After incubating with MG132 for a duration of 6 hours, cells overexpressing MKRN2 in a stable manner were lysed. Subsequently, immunoprecipitation was performed using an antibody targeting PPP2CA, followed by western blot analysis with an anti-ubiquitin antibody. Significance levels denoted as follows: ****p < .0001, ***p < .001, **p < .01, and *p < .05.

Both endogenous and exogenous immunoprecipitation experiments confirmed the interaction between MKRN2 and PPP2CA (Fig. 5A-5D). Immunofluorescence analysis further corroborated this finding (Fig. 5E). Functional analysis of MKRN2 revealed a significant association between protein ubiquitination and degradation (Fig. 5F). To validate this, we conducted protein half-life experiments using CHX. The results showed that MKRN2-OE significantly accelerated the degradation of PPP2CA (Fig. 5G-5J). Following chloroquine treatment, PPP2CA expression in MKRN2-OE cells was notably lower than in control cells, whereas MG132 treatment resulted in similar PPP2CA expression levels in both MKRN2-OE and control cells (Fig. 5K). MKRN2 enhanced the ubiquitination of PPP2CA (Fig. 5L). These findings suggest that MKRN2 regulates PPP2CA expression primarily via the ubiquitin-proteasome pathway. Both endogenous and exogenous immunoprecipitation experiments confirmed the interaction between MKRN2 and PPP2CA (Fig. 5A-5D). IF analysis further corroborated this connection (Fig. 5E). Functional analysis of MKRN2 revealed a significant association between protein ubiquitination and degradation (Fig. 5F). As validation, we conducted protein half-life experiments using CHX and found that MKRN2-OE significantly accelerated PPP2CA degradation (Fig. 5G-5J). Following chloroquine treatment, PPP2CA expression in MKRN2-OE cells was notably lower than in control cells, whereas MG132 treatment led to similar PPP2CA expression levels across both MKRN2-OE and control cells (Fig. 5K). Moreover, MKRN2 enhanced PPP2CA ubiquitination (Fig. 5L). These findings suggest that MKRN2 regulates PPP2CA expression primarily via the ubiquitin-proteasome pathway.

**Figure 6:**
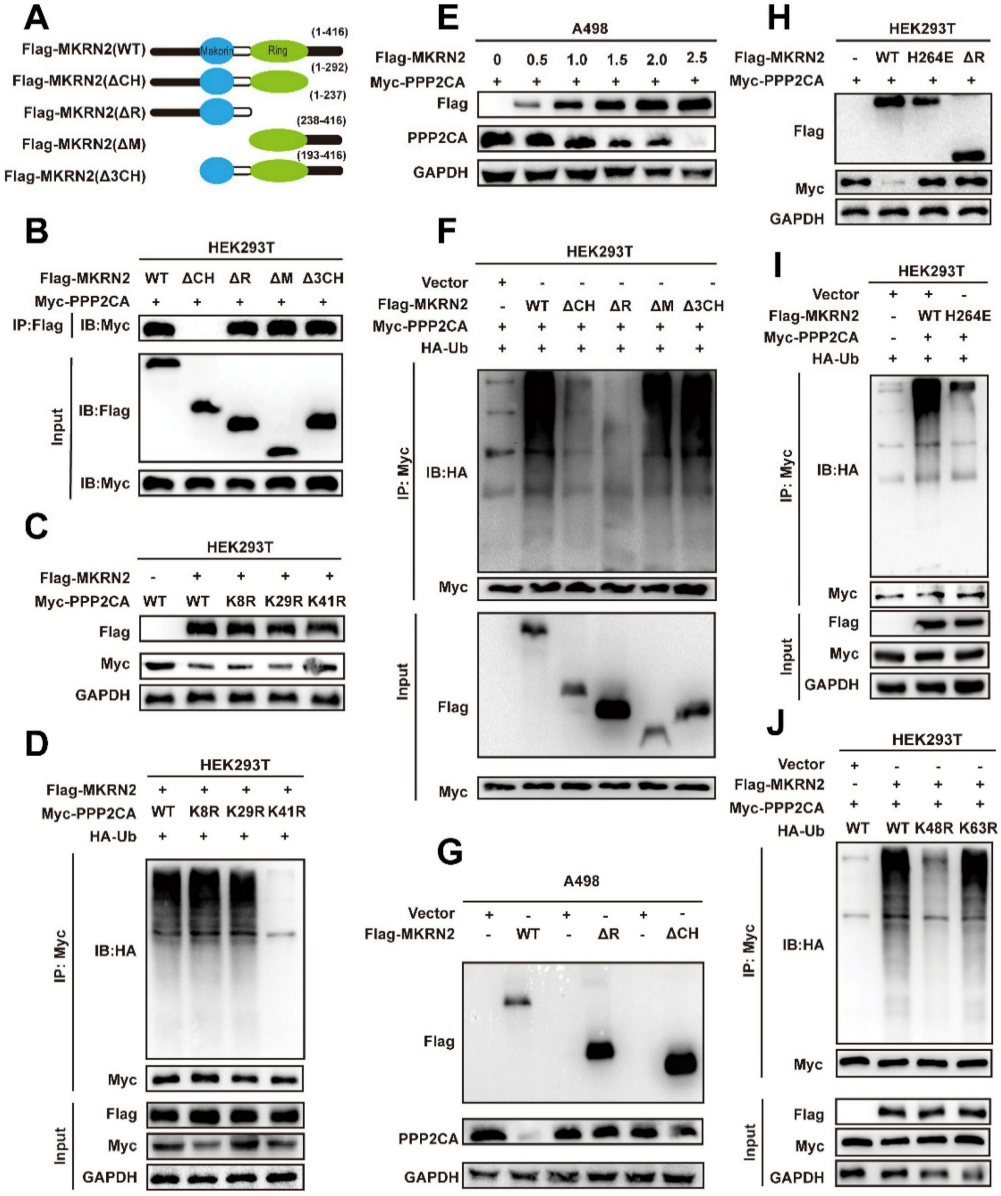
** MKRN2 mediates K48-linked poly-ubiquitination of PPP2CA at its lysine-41 (K41) residue.** (A) Illustrated are schematic depictions of human MKRN2(WT) and its truncation mutants. (B) A498 cells were transfected with plasmids encoding Flag-tagged MKRN2 for 48h. Cell lysates were analyzed by western blot with indicated antibodies. (C) HEK293T cells were co-expressed with wild-type and lysine residual mutated Myc-tagged PPP2CA plasmids for 48h prior to western blot analyses. (D) HEK293T cells were co-expressed with wild-type and lysine residual mutated Myc-tagged PPP2CA plasmids for 48h prior to ubiquitination analyses. (E) A498 cells were transfected with indicated plasmids or a control vector for 48h prior to WB analyses. (F) Wild-type and lysine residual mutated Myc-tagged PPP2CA plasmids were individually transfected into A498 cells, with or without Flag-tagged MKRN2. After 48h prior to ubiquitination analyses. (G)A498 after co-transfection with the indicated constructs. After 48h, cell lysates were analyzed by western blot with indicated antibodies. (H) HEK293T cells were transfected with plasmids encoding Myc-tagged PPP2CA, along with a plasmid encoding Flag-tagged wild-type MKRN2 or MKRN2 mutants (H264E, ΔR). After 48h, cell lysates were analyzed by western blot with indicated antibodies. (I) MKRN2 instead of MRKN2 (H264E) promoted the polyubiquitination of Myc-tagged PPP2CA *in vitro*. (J) HEK293T cells were co-expressed Flag-tagged MKRN2 or a control vector with Myc-PPP2CA and wild type (WT) or mutant HA-tagged ubiquitin (HA-Ub, HA-Ub K48R, HA-Ub K63R) for 48h.

We designed MKRN2 truncations to identify specific domains responsible for binding with PPP2CA (Fig. 6A). We co-expressed PPP2CA with a Myc-tagged protein and MKRN2 with a FLAG-tagged protein in 293T cells (Fig. 6B). Exogenous immunoprecipitation experiments showed that the two proteins did not interact when the N-terminal (ΔCH) was absent (Fig. 6B). Bioinformatic analysis, focusing on conserved lysine residues and ubiquitination sites across species, identified K41R as a key site for maintaining PPP2CA stability, while mutations at other sites had no significant effect on PPP2CA (Fig. 6C). Additionally, PPP2CA ubiquitination levels decreased at the K41R mutation site, while ubiquitination at other mutation sites remained unchanged (Fig. 6D). MKRN2-OE in A498 cells occurred concurrently with a gradual downregulation of endogenous PPP2CA (Fig. 6E). Consistently, PPP2CA ubiquitination was markedly lower when MKRN2ΔR or ΔCH mutants were overexpressed, in contrast to the effects of wild-type MKRN2 -OE (Fig. 6F). Moreover, both MKRN2ΔR and ΔCH were poor facilitators of PPP2CA degradation in renal cancer cells (Fig. 6G).

The RING domain of E3 ubiquitin ligases is crucial for their enzymatic function; our findings showed that H264E and ΔR mutants in the RING domain completely abolished PPP2CA ubiquitination (Fig. 6I), stabilizing PPP2CA (Fig. 6H). These results highlight the importance of the RING and ΔCH domains in MKRN2 binding with PPP2CA and functioning as a ubiquitin ligase. We also found that ectopic MKRN2 expression significantly stimulated PPP2CA polyubiquitination, specifically via Lys 48(K48)-linked, but not Lys 63(K63)-linked, polyubiquitin chains, supporting proteasome-mediated degradation (Fig. 6J) 24, 25.

### MKRN2 destabilizes β-Catenin proteins that are in complex with PPP2CA

**Figure 7:**
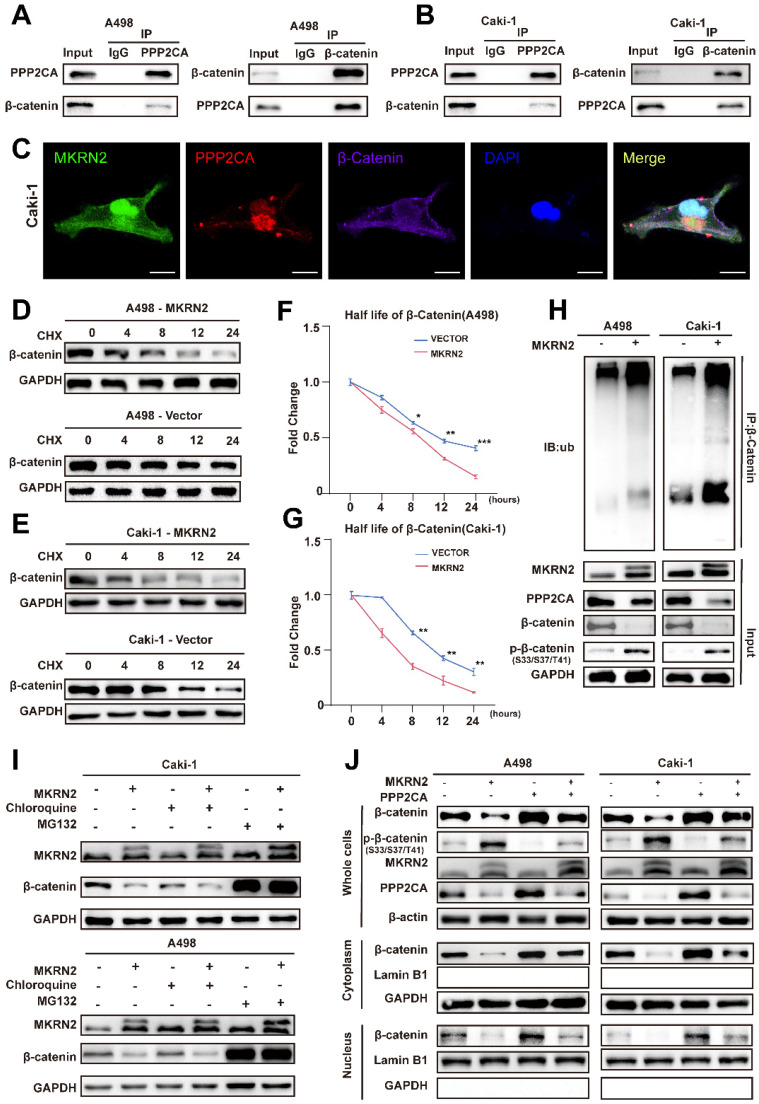
** MKRN2 mediates the poly-ubiquitination and destabilization of PPP2CA-binding partner β-Catenin.** (A, B) Co-IP assays were conducted to ascertain the endogenous interaction between PPP2CA and β-Catenin in A498 and Caki-1 cells. (C) Presented are representative confocal microscopic images displaying the colocalization of MKRN2, PPP2CA, and β-Catenin in A498 cells. (D, E) ccRCC cells with stable MKRN2-OE were subjected to cycloheximide (CHX) treatment at specified time intervals. Subsequently, cells were collected, and PPP2CA protein expression was assessed via western blotting (n = 3). (F, G) Quantitative analysis was performed to determine the relative protein level of PPP2CA. (H) Cells exhibiting stable MKRN2-OE were lysed and subjected to immunoprecipitation utilizing an antibody against β-Catenin, subsequently analyzed by western blotting with an anti-ubiquitin antibody. After incubating with MG132 for a duration of 6 hours, cells overexpressing MKRN2 in a stable manner were lysed. Subsequently, immunoprecipitation was performed using an antibody targeting β-Catenin, followed by western blot analysis with an anti-ubiquitin antibody. (I) ccRCC cells with stable MKRN2 -OE were treated with vehicle (DMSO), MG132 (20 × 10-6 M), or chloroquine (50 × 10-6 M) for 12 hours. Western blot analysis was employed to evaluate the protein level of PPP2CA. (J) Western blot analysis was performed on p‑β-Catenin and β-Catenin protein levels derived from whole‑cell, nuclear, and cytoplasmic extracts in A498 and Caki-1 cells stably transfected with the designated lentiviruses. GAPDH, α-Tubulin, and Lamin B1 were employed as loading controls.

β-Catenin is a key factor in Wnt-mediated transcriptional cascade and next, we addressed whether β-Catenin can be a mediator of PPP2CA biological functions. We first performed Co-IP tests in A498 and Caki-1 cells to validate the mutual binding between β-Catenin and PPP2CA proteins (Fig. 7A-7B); and then conducted co-focal fluorescence assay to confirm their co-localization (Fig. 7C). Nevertheless, PPP2CA-KD would reduce the transcripts of Wnt signature genes but not β-Catenin (Fig. S6J-S6K), indicating PPP2CA regulates β-Catenin protein but not mRNA. Importantly, protein stability assays demonstrated that MKRN2 reduced β-Catenin stability (Fig. 7D-7G). In accordance, the degradation of β-Catenin proteins was associated with typical ubiquitination signals that would be enhanced by MKRN2 (Fig. 7H-7I). We monitored β-Catenin total and subcellular protein expression in A498 and Caki-1 cells and found nuclear localization of β-Catenin proteins and indeed, β-Catenin phosphorylation inversely correlated with its total protein levels (Fig. 7J).

### MKRN2 repressed the progression of ccRCC *in vivo* via the PPP2CA-β-Catenin-Wnt pathway

**Figure 8:**
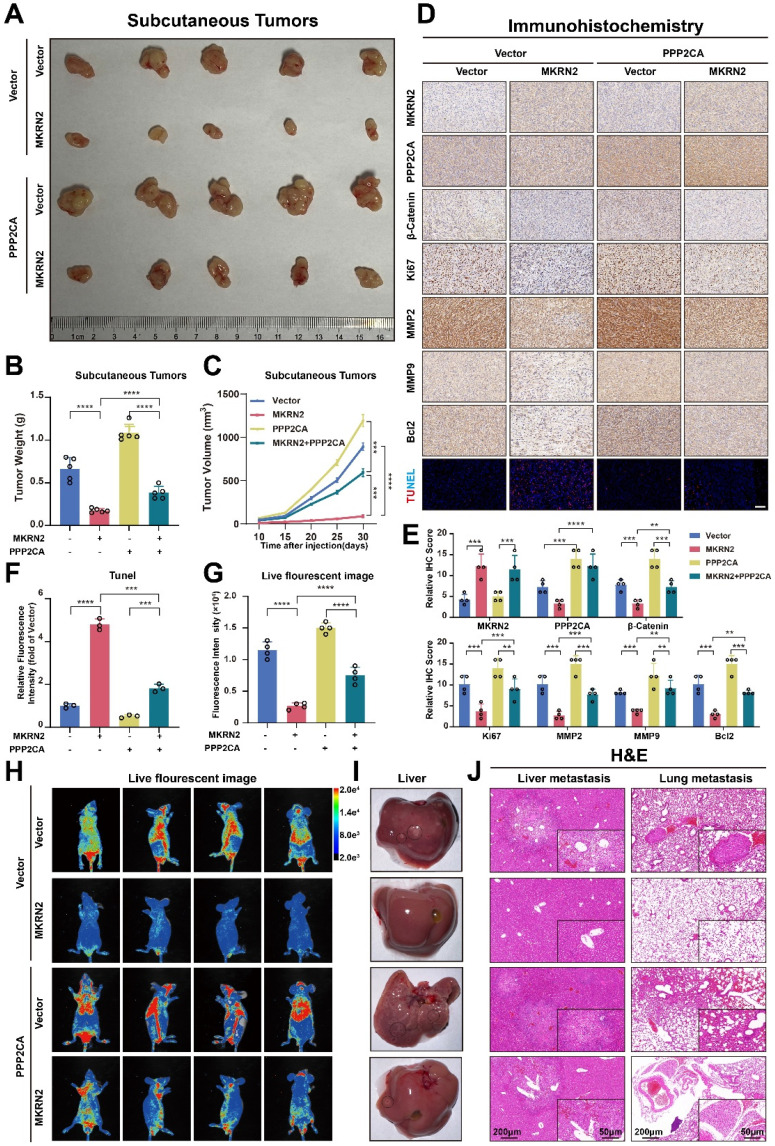
**MKRN2 and PPP2CA have opposite effects on Wnt activation and ccRCC progression in mouse xenografts.** The MKRN2-PPP2CA-β-Catenin axis exhibited inhibitory actions on tumor advancement and concurrently facilitated apoptotic events in tumor cells within the *in vivo* setting. (A) Depicted are representative images of subcutaneous tumors isolated from nude mice. (B) The tumors were excised and weighed after euthanizing the mice (n = 5), with statistical analysis conducted via t-test. (C) Tumor dimensions were assessed every five days, culminating in a final measurement on day 30 (n = 5), with statistical analysis conducted via t-test. (D-E) IHC staining was performed for MKRN2, PPP2CA, β-Catenin, MMP2, MMP9, Bcl2, Ki67, and Tunel stain in the subcutaneous tumors (n = 3), with statistical analysis conducted via t-test. (F) The relative fluorescence intensity of Tunel stain in the subcutaneous tumors from the four groups (n = 3), with statistical analysis conducted via t-test. (G) Live fluorescence imaging was undertaken in the metastasis mode for the four groups. (H) Live small animal fluorescent images were captured for the Vector + Vector, MKRN2 + Vector, Vector + PPP2CA, and MKRN2 + PPP2CA groups in the metastasis model (n = 3). (I) Presented is an image of the liver in the four groups. (J) The H&E staining was performed on lung and liver metastases from the four groups, with scale bars indicating 200 μm and 50 μm. Significance levels denoted as follows: ****p < .0001, ***p < .001, **p < .01, and *p < .05.

**Figure 9:**
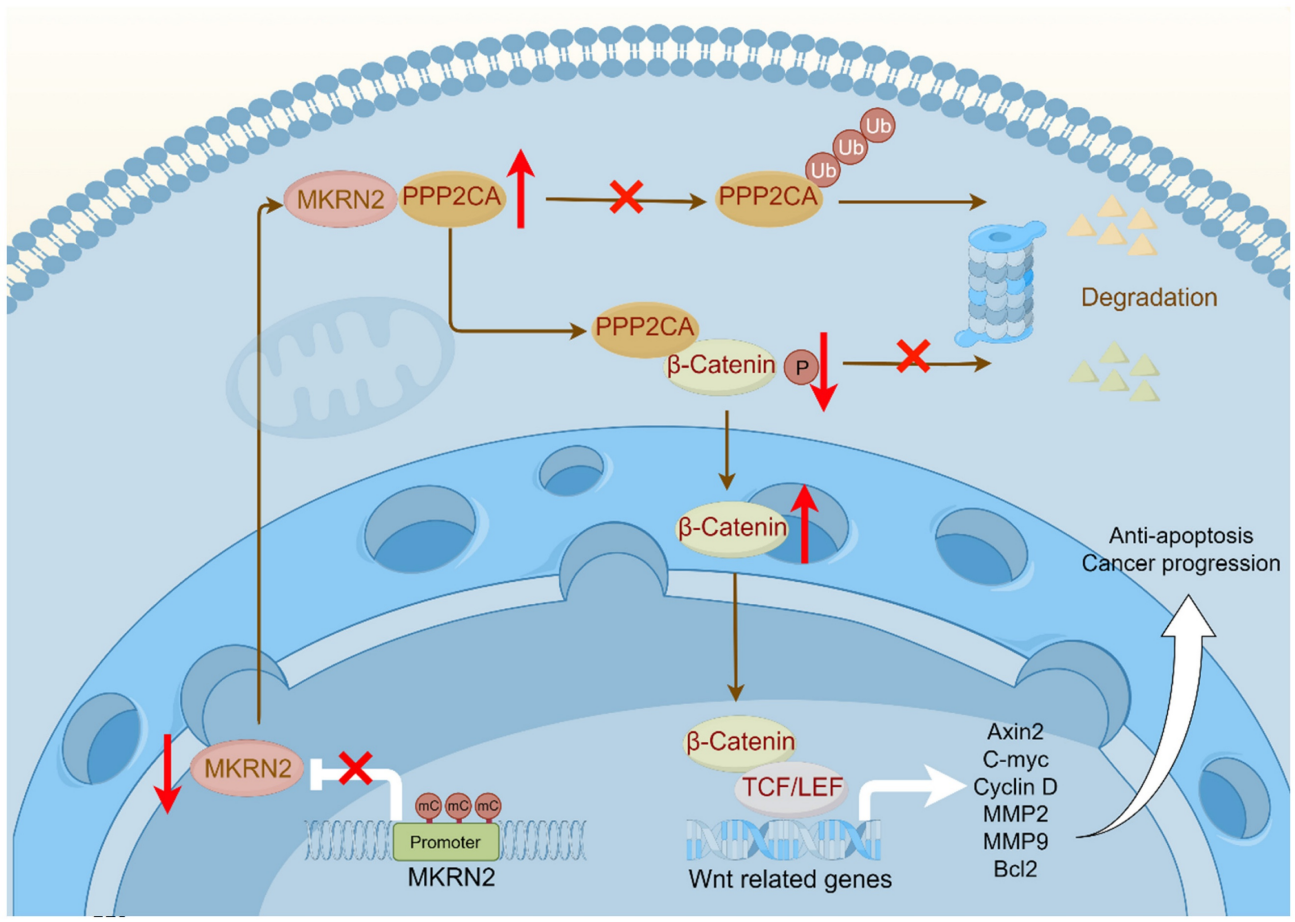
**The illustration depicts a schematic model delineating the operational mechanisms of MKRN2 in clear cell renal cell carcinoma (ccRCC).** To summarize, our investigation discerned that MKRN2, acting as a tumor suppressor, undergoes downregulation in ccRCC, a process facilitated by promoter hypermethylation. MKRN2 precisely targets PPP2CA at the K41 residue, instigating a sequence of events involving polyubiquitination and eventual degradation. Consequently, this molecular process impedes the dephosphorylation of the N-terminal segment of β-Catenin, thereby inhibiting the activation of the Wnt signaling pathway.

We next investigated the effects of MKRN2 on the PPP2CA-β-Catenin-Wnt axis *in vivo* using subcutaneous tumor models. As shown, MKRN2-OE markedly inhibited tumor burdens compared to the control vector group, while PPP2CA-OE reversed the growth inhibition upon MKRN2-OE (Fig. 8A-8C). Moreover, PPP2CA-OE antagonized the magnitude of cell apoptosis elicited by MKRN2-OE in subcutaneous tumors and IHC assays showed that the down-regulation of Ki67, MMP2, MMP9, and Bcl2 in the MKRN2-OE group was partially restored by PPP2CA-OE (Fig. 8D-8F). Furthermore, live small-animal fluorescent imaging showed that PPP2CA-OE counteracted the metastatic inhibition conferred by MKRN2-OE, increasing the number of metastatic nodes in the livers and lungs of mice (Fig. 8G-8J). Based on these findings, we propose that MKRN2 and PPP2CA have opposite regulatory effects on β-Catenin protein expression, leading to functional equilibration of the Wnt signaling that mediates ccRCC tumor progression (Fig. 9).

## Discussion

Understanding the nature of this disease is crucial for treatment, in which epigenetics have fundamental significance 26. Epigenetics, a critical area of biomedical research, helps elucidate mechanisms underlying biological processes 27. Among the various epigenetic modifications, DNA methylation is particularly important 28-31. Hypermethylation of the MKRN2 promoter region contributes significantly to reduced gene expression. Extensive research on ccRCC has shown how gene methylation affects its initiation and progression. Tumor related signaling pathways promote the development of tumors 32-34. An accumulating body of evidence underscores the role of aberrantly activated Wnt signaling in driving critical cancer processes, including cell proliferation, migration, and metastasis 35-37. This study delineates a negative association of MKRN2 with Wnt activation and ccRCC progression. In tumor cells, MKRN2 promoter is marked by hypermethylation that lowers its gene transcription. Mechanistically, in protein interacting complex, MKRN2 promotes PPP2CA ubiquitination and protein destabilization, resulting β-Catenin protein phosphorylation and degradation, then inactivating of the Wnt pathway, leading to cell apoptosis and anti-tumorigenesis (Fig. 9).

Ubiquitination is a post-translational modification, essential for maintaining cellular protein homeostasis 37-39. The discernment of specificity within the ubiquitin system is governed by E3 ligases, which selectively designate proteins for ubiquitination 40, 41. E3 ligases can designate target proteins by recognizing a distinct peptide motif known as a degron within the substrate 42. Notably, MKRN2 was observed to obstruct the progression of lung cancer through ubiquitin-mediated degradation of PI3Kp85a 11. Previous study has shown that MKRN1/MKRN2 can inhibit renal cancer progression by regulating P53 43. Our current study represents an inaugural report delineating the role of MKRN2 in restraining ccRCC progression and promoting its apoptosis through PPP2CA-dependent deactivation of Wnt signaling. Our work presents the first evidences that the E3 ligase activity of MKRN2 engages with β-Catenin ubiquitination and degradation, leading to Wnt inactivation in ccRCC settings. In this regulatory process, PPP2CA functions as a mediator that partners with both MKRN2 and β-Catenin proteins, to convey critical signaling cascades.

Through endogenous, exogenous, and *in vitro* ubiquitination assays, we demonstrated that MKRN2 catalyzes the ubiquitination of PPP2CA at the K41 residue via its E3 ligase activity. K48-associated ubiquitinated linkages mainly enter the proteasome pathway for degradation 44. Our findings indicate that PPP2CA ubiquitination leads to increased phosphorylation, in turn deactivating downstream β-Catenin signaling. Protein phosphatase 2A (PP2A) is one of the four principal Ser/Thr phosphatases and comprises a catalytic subunit and a diverse array of additional regulatory subunits, playing a crucial role in Wnt regulation 45, 46. Furthermore, PPP2CA encodes the alpha isoform of the catalytic subunit 47. Ubiquitination of PPP2CA, the catalytic subunit of PP2A, has been implicated in breast cancer growth 48, in contrast, PPP2CA gene is highly expressed in hepatocellular carcinoma tissues and its over-expression correlates with poor prognosis 49. similarly, in neuroblastoma cells, reduction of PPP2CA decreases cell growth 50. Here we demonstrated that MKRN2 catalyzes the K48-linked ubiquitination of PPP2CA proteins, targeting to the proteasome-dependent degradation pathway. In turn, PPP2CA depletion leads to the phosphorylation and down-regulation of β-Catenin proteins, deactivating the canonical Wnt signaling. It was reported that PPP2CA is primarily responsible for the N-terminal dephosphorylation of β-Catenin, leading to β-Catenin protein ubiquitination and degradation by the SCF^β-TrCP^ complex 51. Our experiments similarly showed an interaction between PPP2CA and the N-terminal phosphorylation site of β-Catenin, without significant effect on the C-terminal phosphorylation site, indicating additional phosphotases may engage with the β-Catenin C-terminal dephosphorylation. Since the N-terminal phosphorylation of β-Catenin is associated with its ubiquitination and degradation by SCF^β-TrCP^ 52, future work should assess whether MKRN2 unleashes the cascade of β-Catenin depletion through the SCF^β-TrCP^ module. Taken together, our report grants candidacy to the E3 ubiquitin ligase activity of MKRN2 and the phosphatase activity of its substrate PPP2CA as novel ccRCC therapeutic targets.

## Conclusions

In the current report, we addressed the clinical relevance, functional significance and critical partners of E3 ligase MKRN2 in ccRCC progression. Specifically, MKRN2 functions as a tumor suppressor, by modulating the PPP2CA-β-Catenin axis to inactivate the Wnt pathway.

## Supplementary Material

Supplementary figures and tables.
