# Supplementary material for: E3 ligase MKRN2 destabilizes PPP2CA proteins to inactivate canonical Wnt pathway and mitigates tumorigenesis of clear cell renal cell carcinoma
Source: Int J Biol Sci. 2025 Aug 22;21(12):5361–77. doi: 10.7150/ijbs.107130 (PMC12435490; doi:10.7150/ijbs.107130)
Supplement: Supplementary file 1 — Supplementary figures and tables. [file ijbsv21p5361s1.pdf]

E3 ligase MKRN2 destabilizes PPP2CA proteins to inactivate canonical Wnt pathway and mitigates tumorigenesis of clear cell renal cell carcinoma

Tiexi Yu[a,b,c,*], Weiquan Li[a,b,c,*], Xiangui Meng [a,b,c,*], Hongwei Yuan[a,b,c], Hailong Ruan [a,b,c,#], Wen Xiao [a,b,c,#], Xiaoping Zhang [a,b,c,#]

[*] Tiexi Yu, Weiquan Li, and Xiangui Meng contributed equally to this work

[#] Correspondence:

Hailong Ruan hlruan2010@126.com

Wen Xiao wxuro20@hust.edu.cn

Xiaoping Zhang xzhang@hust.edu.cn

[a] Department of Urology, Union Hospital, Tongji Medical College, Huazhong University of Science and Technology, Wuhan, 430022, China.

[b] Shenzhen Huazhong University of Science and Technology Research Institute, Shenzhen 518000, China.

[c] Institute of Urology, Tongji Medical College, Huazhong University of Science and Technology, Wuhan 430022, China.

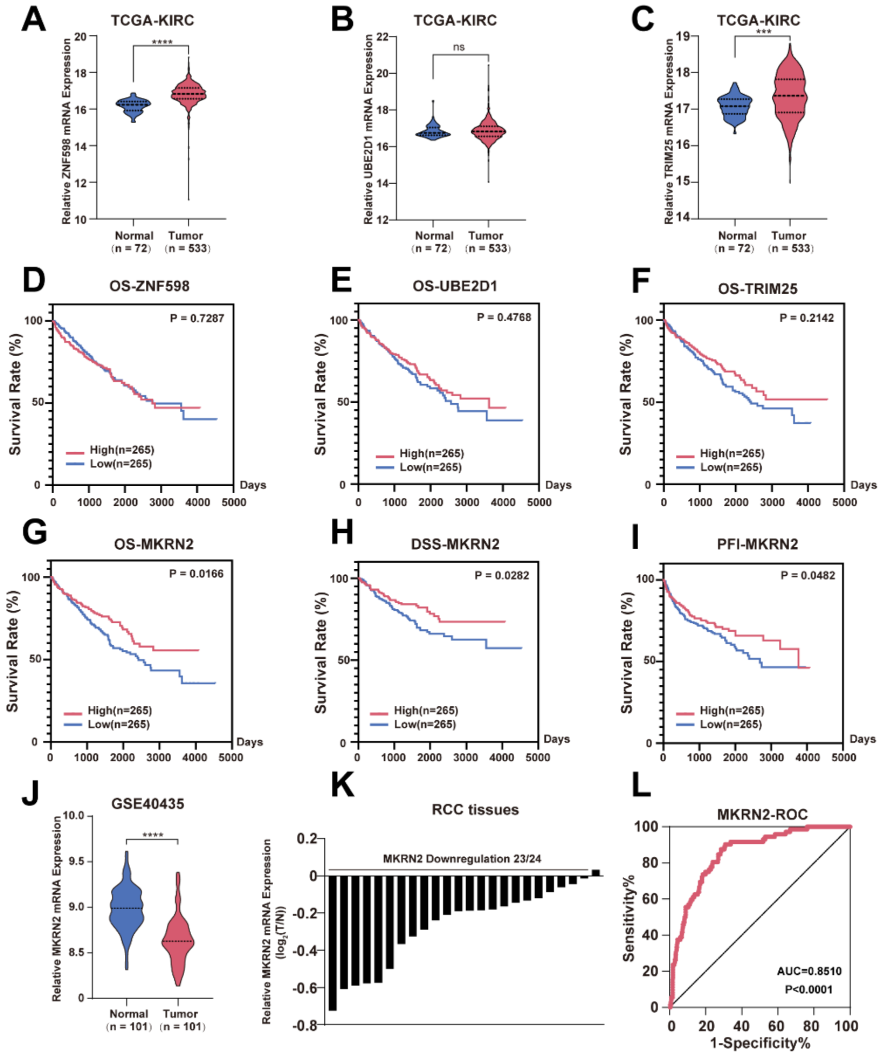

Supplementary Fig. 1： MKRN2 is down-regulated in ccRCC

(A, B, C) The expression level of ZNF598, UBE2D1 and TRIM25 in TCGA-KIRC dataset. (D, E, F, G) The Kaplan-Meier analyses of the association between the overall survival rate of ccRCC patients and the expression levels of ZNF598, UBE2D1, TRIM25 and MKRN2. (H) The disease specific survival of patients in high and low expression level of MKRN2. (I) The progression free interval of patients in n high and low expression level of MKRN2. (J) The expression level of MKNR2 in ccRCC from GSE40435 dataset. (K) The mRNA levels of MKRN2 in ccRCC tissues and paired adjacent normal tissues from 24 patients. (L) ROC curve of MKRN2 in ccRCC from TCHA-KIRC dataset.

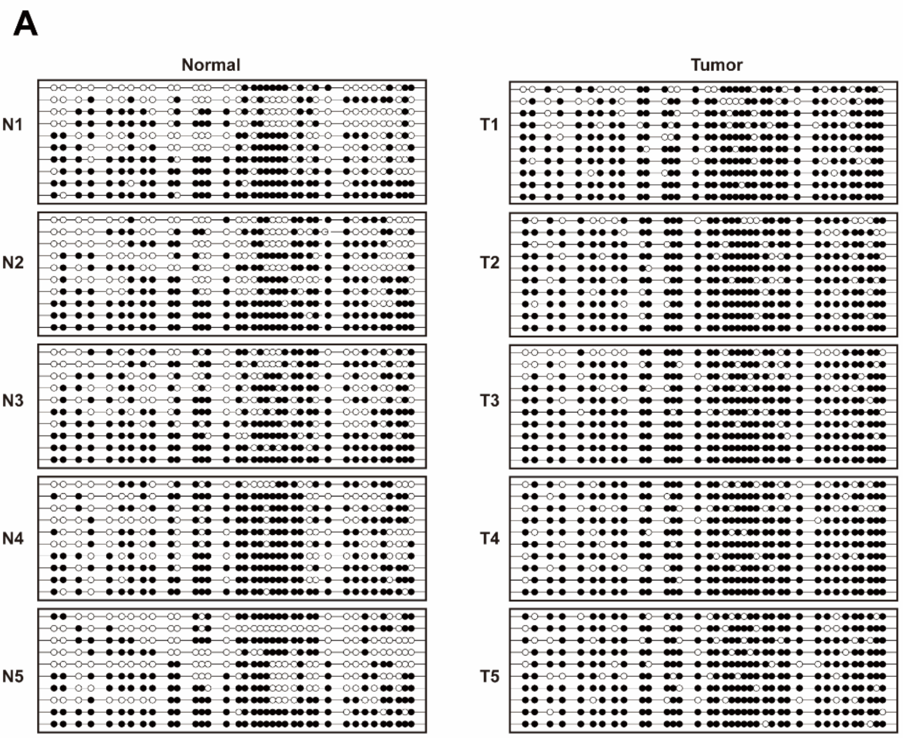

Supplementary Fig. 2: DNA methylation caused the low expression of MKRN2 in ccRCC.

(A) BSP results of MKRN2 methylation status in adjacent tissues (N) and ccRCC tissues (T).

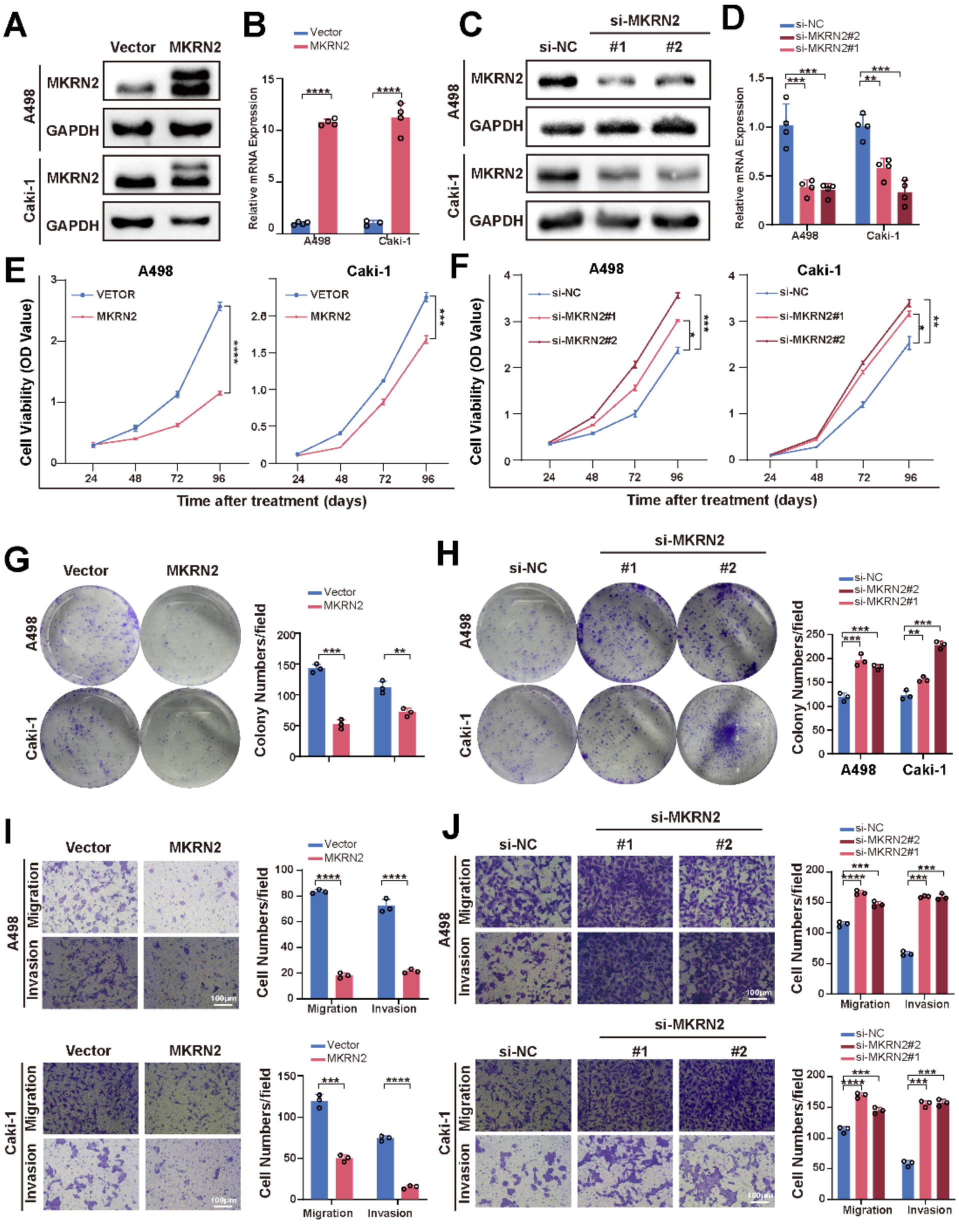

Supplementary Fig. 3: MKRN2 demonstrated a suppressive effect on the in vitro progression of ccRCC. (A, B, C, D) MKRN2-overexpressing or MKRN2-knockdown ccRCC cell lines were established by transfecting overexpressing lentivirus and siRNA. (E, F) Cell growth curves were generated through CCK8 assays for the specified cells (n = 3). (G, H) Colony formation assays were conducted for the indicated ccRCC cells (n = 3). (I, J) Migration and invasion assays were performed for the specified ccRCC cells (n = 3). Significance levels denoted as follows: ****p < .0001, ***p < .001, **p < .01, and *p < .05.

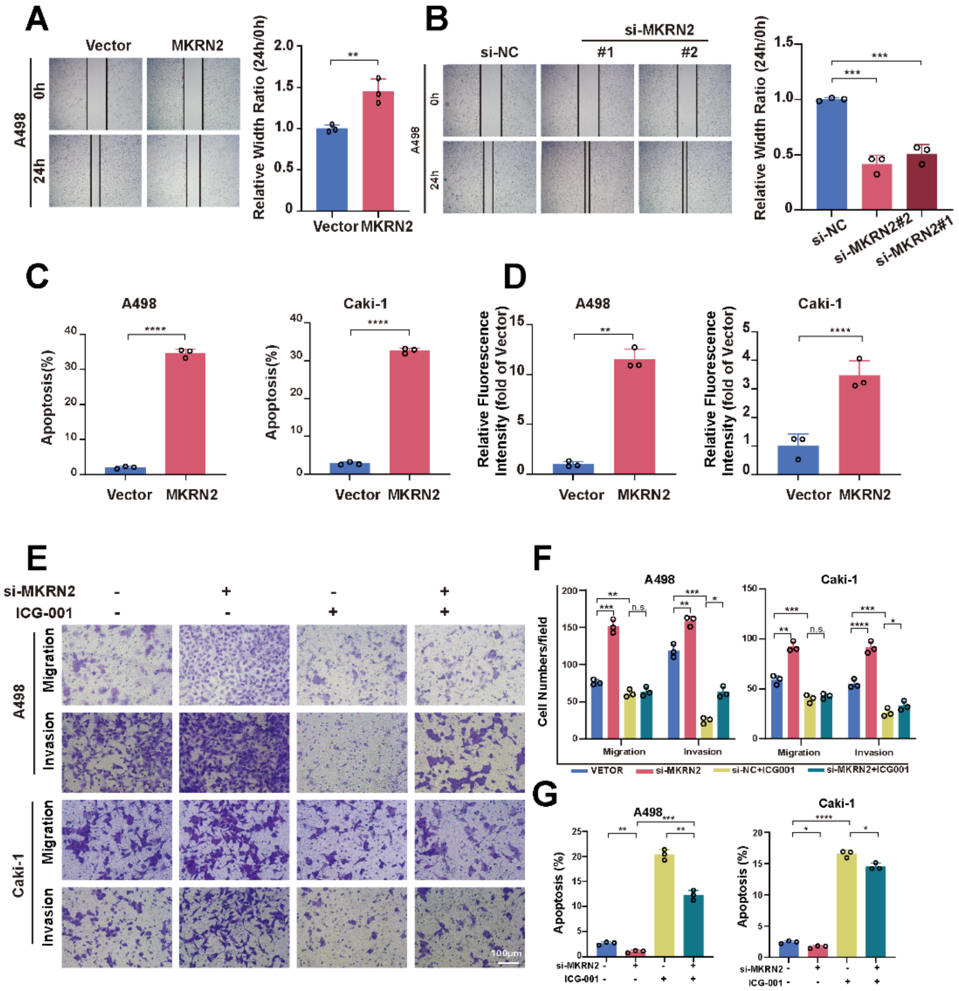

Supplementary Fig. 4: MKRN2 repressed Wnt signaling pathways activation and tumor progression.

(A, B) Wound healing assay were generated for the A498 cells with MKRN2 overexpression and knock down (n = 3). (C) The apoptosis rate for the cells with MKRN2-overexpresion (n = 3). (D) The relative fluorescence intensity of TUNEL for the cells with MKRN2-overexpresion (n = 3). (E, F) The relative cell number of the migration and invasion assays for the specified cells (n = 3). Cells with MKRN2 knockdown were treated with 10 μM ICG-001 (an inhibitor of β-catenin/TCF) or DMSO. (G) The apoptosis rate for the cells for the specified cells (n = 3). Significance levels denoted as follows: ****p < .0001, ***p < .001, **p < .01, and *p < .05.

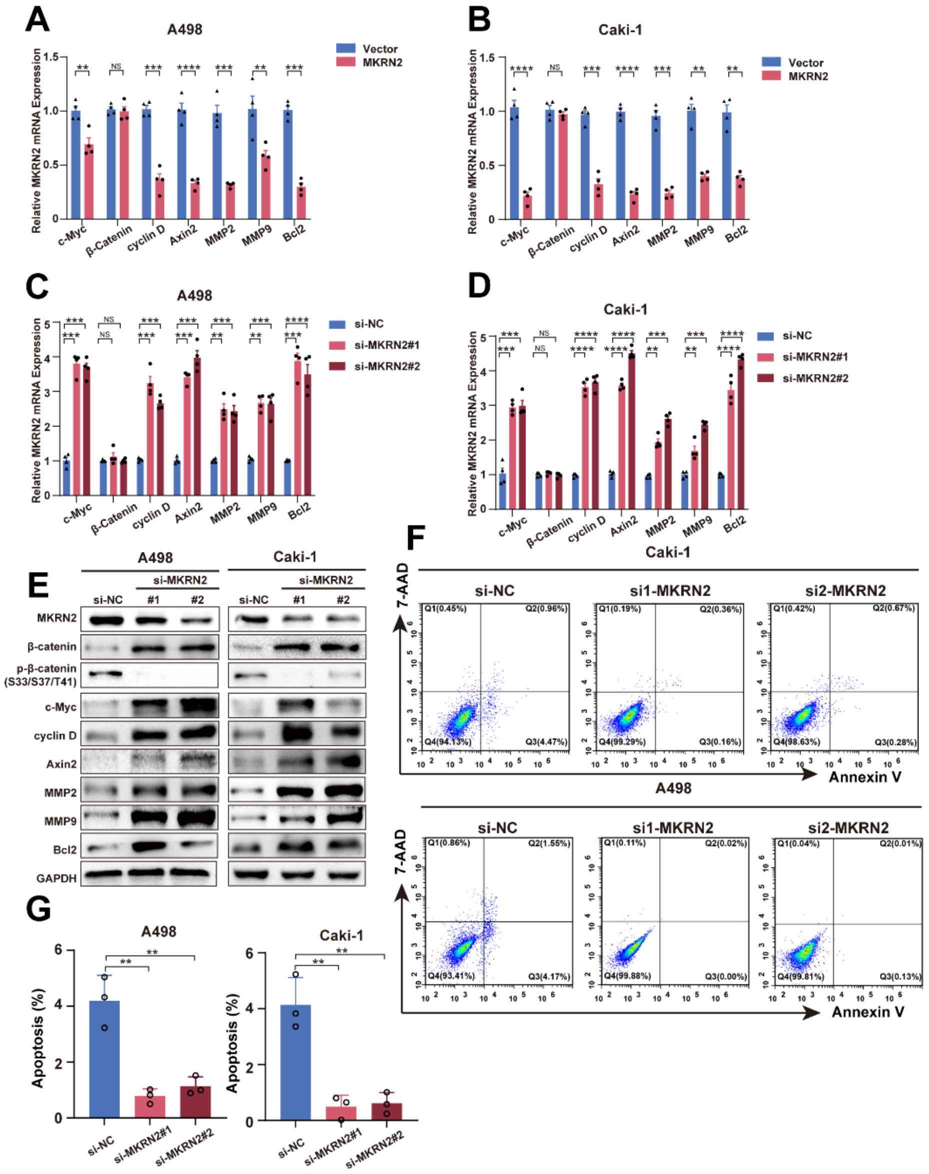

Supplementary Fig. 5: MKRN2 inhibited the activation of Wnt signaling pathway.

(A, B) Relative mRNA expression of wnt related genes for the cells with MKRN2-overexpression (n = 4). (C, D) Relative mRNA expression of wnt related genes for the cells with MKRN2-knockdown (n = 4). (E) Protein expression of wnt related genes for the cells with MKRN2-knockdown (n = 3). (F) Flow cytometry analysis was employed to determine the proportion of apoptotic cells in samples with MKRN2 knockdown compared to control cells (n = 3). (G) The apoptosis rate in the MKRN2 knock down cell lines (n = 3). Significance levels denoted as follows: ****p < .0001, ***p < .001, **p < .01, and *p < .05.

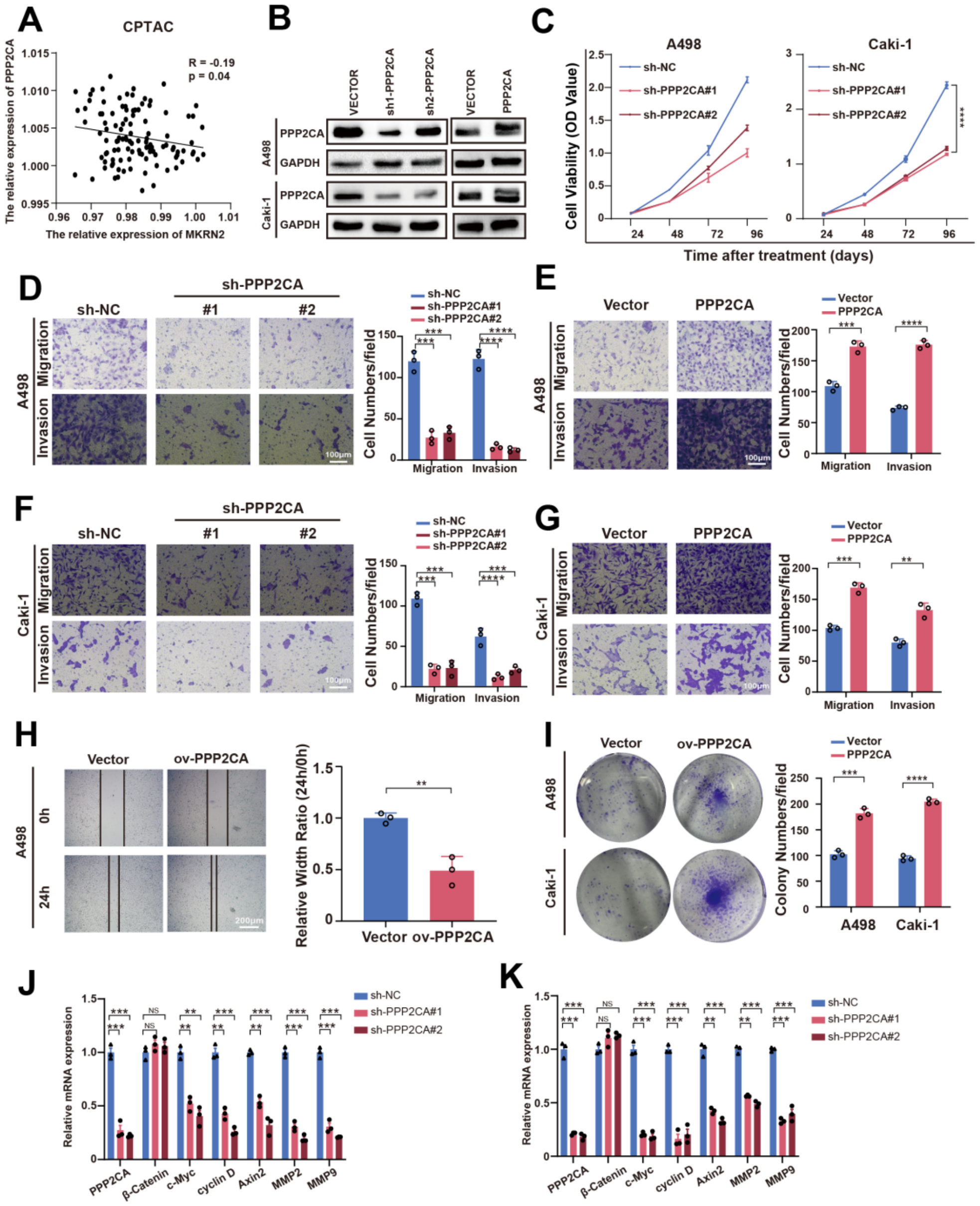

Supplementary Fig. 6: PPP2CA played an oncogenic role in ccRCC.

(A) MKRN2 proteins had a negative correlation with PPP2CA proteins in ccRCC. (B) PPP2CA-overexpressing or PPP2CA-knockdown ccRCC cell lines were established by transfecting overexpressing lentivirus and siRNA. (C-G) CCK8 and Transwell assay for the indicated cells. And

the relative cell number of the migration and invasion assays for the cells with PPP2CA-overexpression or sh-PPP2CA (n = 3). (H) Wound healing assay for the cells with PPP2CA-overexpression (n = 3). (I) Colony assay for the cells with PPP2CA-overexpression (n = 3). (J)-(K) mRNA level of Wnt signing effected by sh-PPP2CA. Significance levels denoted as follows: ****p < .0001, ***p < .001, **p < .01, and *p < .05.

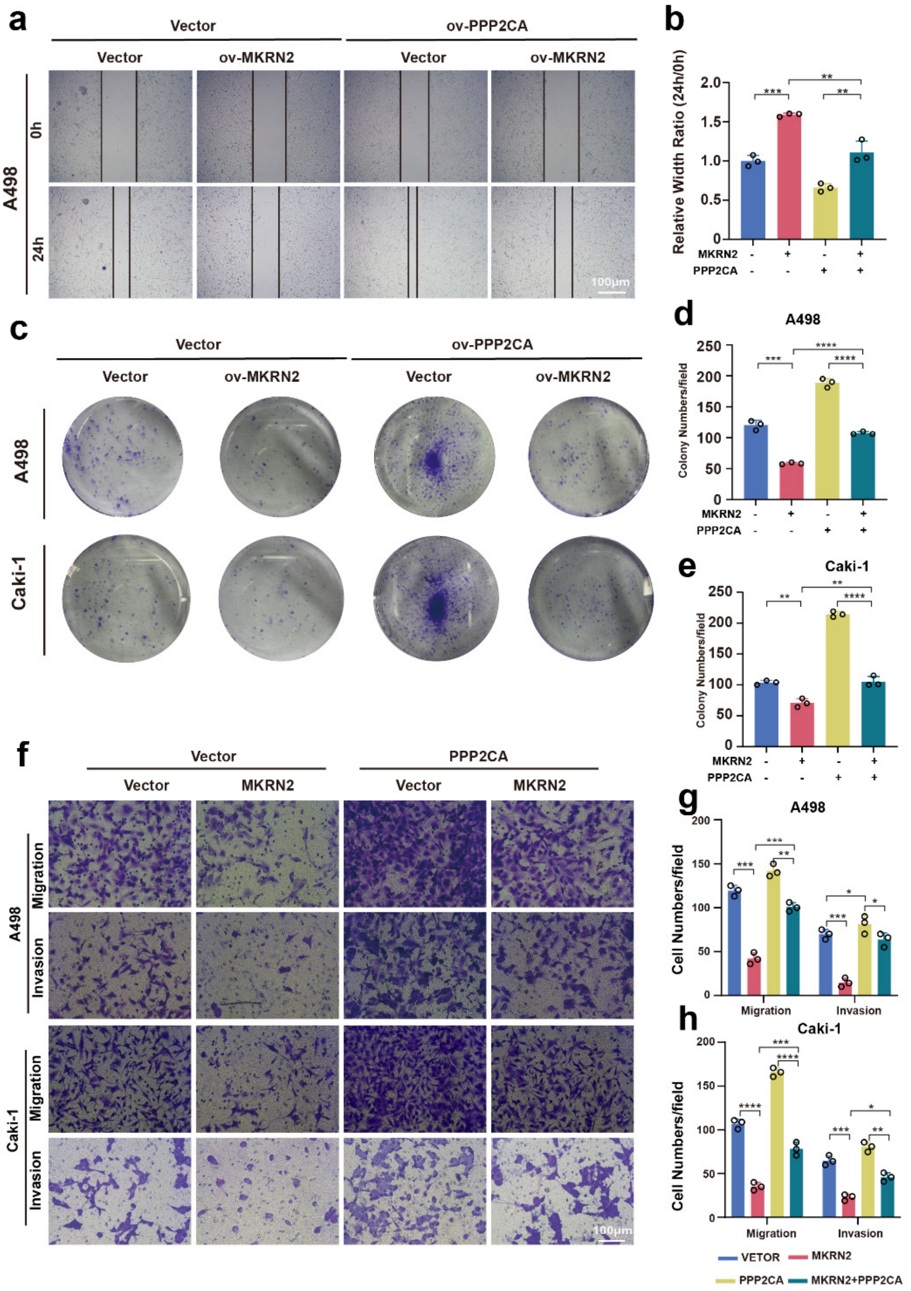

Supplementary Fig. 7: MKRN2 inhibited ccRCC progression through PPP2CA in vitro.

(A) Wound healing assay were generated for the specified cells. (B) The relative width ratio of wound healing assay for for the specified cells. (n = 3). (C) Colony assay for the specified cells. (D,

E) The colony numbers for the cells with PPP2CA-overexpression (n = 3). (F, G, H) Migration and invasion assays were conducted for the indicated ccRCC cells (n = 3) (Magnification: 200×). Significance levels denoted as follows: ****p < .0001, ***p < .001, **p < .01, and *p < .05.

Supplementary table 1

**First antibodies**:	**Brand**	**Cat No.**	**Dilution**
anti-DDDDK-Tag	Abclonal	AE169PM	1:1000
anti-Myc-Tag	Abclonal	AE010	1:1000
anti-Phospho-B-Catenin-S29/33/37/T41	Abclonal	AP1076	1:500
anti-Phospho-B-Catenin-S552	Abclonal	AP1315	1:1000
anti-BcI-2	Abclonal	A19693	1:1000
anti-MMP2	Abclonal	A19080	1:1000
anti-MMP9	Abclonal	A26079	1:1000
anti-PPP2CA	Abclonal	A6702	Wb: 1:1000<br>IF: 1:50
anti-β-Catenin	Proteintech	51067-2-AP	Wb:1:1000<br>IF: 1:100
anti-β-Catenin	Abclonal	A19657	1:2000
anti-MKRN2	Santa Cruze	sc-101118	Wb: 1:100<br>IF: 1:100
anti-Axin2	Abclonal	A2513	1:2000
anti-PPP2R1A	Abclonal	A18571	1:1000
anti-Cyclin D	Abclonal	A19038	1:1000
**Secend antibodies:**			
HRP Goat Anti-Rabbit IgG (H+L)	Abclonal	AS014	1:3000
HRP Goat Anti-Mouse IgG (H+L)	Abclonal	AS003	1:3000
Dylight 488, Goat Anti-Rabbit IgG	abcam	ab96899	IF: 1:100
Dylight 549, Goat Anti-Rabbit IgG	abcam	ab96885	IF: 1:100
Dylight 488, Goat Anti-Mouse IgG	abcam	ab96879	IF: 1:100
Dylight 549, Goat Anti-Mouse IgG	abcam	ab96881	IF: 1:100
CoraLite647-conjugated Goat AntiMouse lgG	Proteintech	SA00014-10	IF: 1:200

Supplementary table 2

Symbol	Sequence	NCBI accession numbers	Lengths

MKRN2-F	AGGAAGTCAGTGCCTATTCTCA	NM_014160.5	111
MKRN2-R	TGGTCATATCTGCACCGAGTT		
PPP2AC-F	CAAAAGAATCCAACGTGCAAGAG	NM_002715.4	211
PPP2AC-R	CGTTCACGGTAACGAACCTT		
BCL2-F	GGTGGGGTCATGTGTGTGG	NM_000657.3	89
BCL2-R	CGGTTCAGGTACTCAGTCATCC		
KI67-F	ACGCCTGGTTACTATCAAAAGG	NM_002417.5	209
KI67-R	CAGACCCATTTACTTGTGTTGGA		
MMP9-F	TGTACCGCTATGGTTACACTCG	NM_004994.3	97
MMP9-R	GGCAGGGACAGTTGCTTCT		
MMP2-F	TACAGGATCATTGGCTACACACC	NM_004530.6	90
MMP2-R	GGTCACATCGCTCCAGACT		
CCND1-F	GCTGCGAAGTGGAAACCATC	NM_053056.3	135
CCND1-R	CCTCCTTCTGCACACATTTGAA		
CTNNB1-F	AAAGCGGCTGTTAGTCACTGG	XM_047447479.1	215
CTNNB1-R	CGAGTCATTGCATACTGTCCAT		
CMYC-F	GGCTCCTGGCAAAAGGTCA	NM_002467.6	119
CMYC-R	CTGCGTAGTTGTGCTGATGT		
